# Patellar tendinopathy in young elite soccer– clinical and sonographical analysis of a German elite soccer academy

**DOI:** 10.1186/s12891-017-1690-2

**Published:** 2017-08-08

**Authors:** Gerrit Bode, Thorsten Hammer, N. Karvouniaris, M. J. Feucht, L. Konstantinidis, N. P. Südkamp, A. Hirschmüller

**Affiliations:** 1 Freiburg University Hospital, Clinic of Orthopedic Surgery and Traumatology, Hugstetter Str. 55, 79098 Freiburg, Germany; 2 Altius Swiss Sportmed Center Ag, Rheinfelden, Switzerland

**Keywords:** Patellar tendinopathy, Jumper’s knee, Soccer, Youth elite players

## Abstract

**Background:**

The prevalence of patellar tendinopathy is elevated in elite soccer compared to less explosive sports. While the burden of training hours and load is comparably high in youth elite players (age < 23 years), little is known about the prevalence of patellar tendinopathy at this age. There is only little data available on the influence of age, the amount of training, the position on the field, as well as muscular strength, range of motion, or sonographical findings in this age group. The purpose of the present study was to examine the above-mentioned parameters in all age groups of a German youth elite soccer academy.

**Methods:**

One hundred nineteen male youth soccer players (age 15,97 ± 2,24 years, height 174, 60 ± 10,16 cm, BMI 21, 24 ± 2,65) of the U-13 to U-23 teams were part of the study. Data acquisition included sport specific parameters such as footwear, amount of training hours, leg dominance, history of tendon pathologies, and clinical examination for palpatory pain, indurations, muscular circumference, and range of motion.

Subjective complaints were measured with the Victorian Institute of Sport Assessment Patellar (VISA-P) Score. Furthermore, sonographical examinations (Aplio SSA-770A/80; Toshiba, Tokyo, Japan) with 12-MHz multifrequency linear transducers (8–14 MHz) of both patellar tendons were performed with special emphasis on hyper- and hypo echogenic areas, diameter and neovascularization.

**Results:**

The prevalence of patellar tendinopathies was 13.4%. Seventy-five percent of the players complained of pain of their dominant leg with onset of pain at training in 87.5%. The injured players showed a medium amount of 10.34 ± 3.85 training hours and a medium duration of symptoms of 11.94 ± 18.75 weeks. Two thirds of players with patellar tendinopathy were at the age of 15–17 (Odds ratio 1.89) while no differences between players of the national or regional league were observed.

In case of patellar tendinopathy, VISA-P was significantly lower in comparison to healthy players (mean ± SD 76.80 ± 28.56 points vs. 95.85 ± 10.37). The clinical examination revealed local pain at the distal patella, pain at stretching, and thickening of the patellar tendon (*p* = 0.02).

The mean tendon diameter measured 2 cm distally to the patella was 4.10 ± 0.68 mm with a significantly increased diameter of 0.15 mm in case of an underlying tendinopathy (*p* = 0.00). The incidence of hypo-echogenic areas and neovascularizations was significantly elevated in players with patellar tendon syndrome (PTS) (*p* = 0.05).

**Conclusion:**

The prevalence of patellar tendinopathy in youth elite soccer is relatively high in comparison to available data of adult players. Especially players at the age of 15 to 17 are at considerable risk. Tendon thickening, hypo-echogenic areas, and neovascularization are more common in tendons affected by PTS.

## Background

Patellar tendinopathy (PT) is a very common overuse injury especially in explosive jumping sports [1], often resulting in substantial morbidity and absence from the sporting field [2, 3].

The prevalence of tendinopathies is not well examined so far. For non-elite adult athletes the prevalence varies between 14.4% and 2.5% for different sports [4]. While a prevalence of up to 28% has been published in volleyball with 40% of professional players sustaining PT syndrome at least once in their career [5], only 2.4% of adolescent professional soccer players sustains PT. However, knee pain, focal tenderness or even acute episodes of PT have a major impact on each player’s development, time off the pitch, and career [6]. While high BMI, tall stature, reduced range of ankle movement, and training frequency were detected as risk factors in volleyball [7], only few studies investigated on PT in professional soccer players. Hägglund et al. observed an incidence rate of 0.12 injuries/1000 h and 2.4% of adult players affected per year [6].

In their cohort, 20 % of tendinopathies were recurrent complaints, which represents the high risk of chronification. Thus, preventing the onset of PT seems to be an important factor. Whereas further epidemiological data of PT in adult elite soccer players differ from 7 to 23% [3, 8], comparable data of youth elite players is rare. Recently, a systematic review by Simpson et al. showed that children and adolescent could also sustain PT with a higher risk in boys [9]. While different overuse injuries in youth elite sports have been investigated in detail like Morbus Osgood-Schlatter with comparable underlying pathologies and risk factors [10] it remains unclear, however, if known risk factors for PT in adults like age, increased BMI, and high training load are also applicable in youth players or if other risk factors are even more important, such as playing surface, position on the field, leg dominance, or history of PT in the past. Furthermore, it remains unknown, which age groups endures an elevated risk of developing PTS and probably require adaption of training contents or specific prevention programs.

Considering diagnostics, ultrasound (US) is a safe and efficient method to detect structural tendon changes [11, 12] and adaptions to sport-specific loading. Athletes with PT often present abnormalities in US such as thickening and hypo-echogenic areas. Even though the presence of abnormalities is not always pathognomonic for PT, several studies have used US in combination with clinical examination. Recently, Visnes et al. published prospective data of morphological tendon changes among 158 elite volleyball players demonstrating that baseline US changes are risk factors for developing symptoms of PT. Until now, the influence of sonographical findings often seen in patients with PT [13] have not been examined in youth elite soccer players.

The purpose of the present study was to evaluate prevalence, risk factors, baseline sonographical and clinical findings including VISA-P score and range of motion in a German youth elite soccer academy.

## Methods

### Study population

A case - control study was performed including 119 male youth elite soccer players of a youth academy of a German first division club (age 15.97 ± 2.24 years, height 174.60 ± 10.16 cm, BMI 21.24 ± 2.65). Membership at the youth academy was the main criterion to include players ranging from U-13 to U-23. Exclusion criteria were familiar hypercholesterolemia, rheumatic diseases, and a history of patellar tendon rupture or prior surgery of the patellar tendon. All subjects took voluntarily part in the study and signed an informed consent form in accordance with the Declaration of Helsinki. For all players under the age of 18, parents were informed in a detailed manner and signed a written informed consent form. The ethics committee of the local university approved the study. Baseline data of all players including age, height, weight, and soccer related data such as type of shoes, weekly amount of training hours, position in the field, leg dominance, and medical history of previous tendinopathies, injuries, medication, and surgeries were documented. Clinical examination included motion of the hip, knee and ankle joint by experienced observers using a goniometer, tendon pain at the proximal patella pool to differentiate PTS from M. Osgood-Schlatter, spindle-shaped thickening, and muscle circumference. Subjective complaints were measured using the VISA-P Score [14]. Further radiological diagnostics e.g. in order to differentiate Sinding-Larsen-Syndrom from PTS were not performed with respect to young age and radiation protection.

Ultrasonography examination was performed after a resting period of at least a 2 h (mean 23.00 ± 23.59 h) in accordance to avoid intratendinous blood flow [15].

**Fig. 1:**
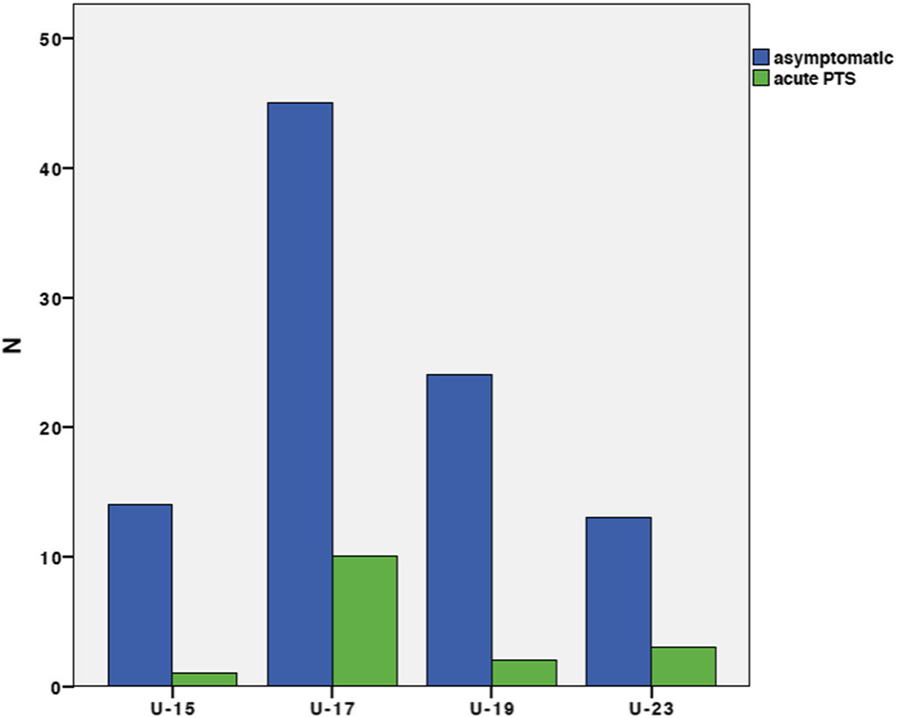
Distribution of players sustaining acute PTS according to the different age groups showing that the U-17 team sustains the highest risk of PTS onset (Odds ratio 1.89)

A high-resolution power-Doppler ultrasonography (Aplio SSA-770A/80; Toshiba, Tokyo, Japan) with 12-MHz multifrequency linear transducers (8–14 MHz) was used for sonographical examination. Players were placed in supine position with both knees passively flexed at 30° and the examiner being blinded to the history of the players. The right and left patellar tendons of each subject were scanned in longitudinal and transverse section while the transducer was placed strictly parallel or orthogonal to the fiber direction as previously described (33,35). B-mode abnormalities of interest included spindle-shaped thickening of the tendon, hyper- and hypoechogenicities, and paratendon thickening (B-mode intensification = 80, penetration depth = 3 cm, focus at 0.5 cm). All pathologic findings were documented in longitudinal and transverse sections. Subsequently, the diameter of the tendon was measured in the longitudinal section. Therefore, the maximum tendon diameter called “true tendon thickness”(39) was measured at a reference point at 2 cm proximally to the patellar tip and at its thickest dimension. The epitendon and paratendon were not included into the measurement. Finally, the tendon was examined for intratendinous micro- vessels using Colour Doppler (Fig. 1; frequency = 10 MHz, pulse repetition frequency = 15.6 kHz, color velocity = 1.2 cmI sj1, color intensity just below the artifact threshold, size of the color box (region of interest, ROI) = 3 cm2 (2 cm _ 1.5 cm)). The pressure of the probe was kept to a minimum to avoid obliteration of small vessels (11). Intratendinous power Doppler flow was graded from 0 to 5, scoring 0 (no vessels visible), 1 (1–2 vessels within the ROI), 2 (3–5 vessels within the ROI), 3 (vessels in up to 30% of the ROI), 4 (vessels in 30%–50% of the ROI), and 5 (vessels in >50% of the ROI) as described earlier [16] in a modified manner of the system described by Gisslén and Alfredson [17]. Additionally, the total number of vessels was documented. All ultrasound scans were performed and analyzed by one experienced and DEGUM certified examiner.

### Statistical analysis

SPSS for Windows (version 21.0; SPSS, Chicago, IL) was used for statistical analysis. Quantitative variables at baseline were expressed as mean ± SD. For statistical evaluation of clinical data including VISA-P and dopplersonographic findings, a student t-test was used. Odds ratio was used to identify players at risk for developing PT. Accordingly, *p* ≤ 0.05 was considered significant.

## Results

In total, 119 players (232 patella tendons) of youth elite soccer players, all competing at the highest leagues were included in the present study. Baseline characteristics and sport specific details are given in Tables 1 and 2. Thirteen players presented symptoms of an acute patellar tendinopathy resulting in a prevalence of 13.60%, while a total of 32 players reported about former history of patellar tendinopathy (27.10%). As players of all youth teams were included, the total amount of training hours ranged from 4.5 to 20 h per week with a mean of 10.35 ± 3.86 h per week. The onset of symptoms took place during training sessions in 85.7% cases while only 14.3% presented PT symptoms at competition for the first time. Local pain and pain in knee flexion where observed during clinical examination in all of the symptomatic cases.

**Table 1:** Baseline descriptive statistics of players’ characteristics

	Min	Max	Mean	Std
Age (years)	10.00	23.00	15.94	2.24
Height (cm)	138	193.00	174.64	10.17
Weight (kg)	30	100.00	65.56	13.5
BMI	14.54	26.85	21.23	2.65
Training units/week	3	10.00	5.43	1.88
Training h/week	4.5	20.00	10.35	3.86

**Table 2:** Soccer specific values and jumper’s knee prevalence

	Number	Percent
U-17	56	47.5
U-19	27	22.90
U-23	16	13.60
Total	119	
Shoes with cleats	94	79.70
Shoes without cleats	5	3.40
Both	15	4.20
Goalkeeper	15	12.70
Central Defender	17	14.40
Lateral Defender	15	12.70
Midfield	18	15.30
Offense	43	36.40
Inlays in Soccer shoes	5	4.20
Inlays in all shoes	13	11.00
No inlays	92	78.00
Left leg dominance	72	61.00
Right leg dominance	39	33.10
Acute Patellar tendinopathy	13	13.60
History of patellar tendinopathy	32	27.10

While none of the sport specific parameters like position, field surface, shoes, insoles, or leg dominance as well as height and weight had any influence on the occurrence of PT, players of the U-17 underwent the highest risk of developing PTS (Odds ratio 1.89) Fig. 1.

Significantly increased scores in all VISA-P subscales and total VISA-P were measured in players with PT (Table 2) compared to players not affected by PTS.

**Fig. 2:**
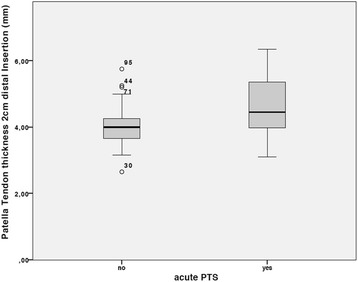
Measurement of patellar tendon thickness at the thickest aspect of the tendon with significant thickening in tendons with acute PTS

An overview of US findings is given in Table 3. The incidence of spindle-shaped thickening and hypo-echogenic areas was significantly higher in players with PT (*p* = 0.001). Furthermore, the patellar tendon was significantly thicker in symptomatic players with a medium diameter of 4.58 ± 0.98 in comparison to 4.00 ± 0.53 at the thickest part (*p* = 0.001) Fig. 2.

**Table 3:** Results of ultrasound examination demonstrating high prevalence of structural changes

	*N* = 232	Percent
Tendon thickening	9	7.60
Hypo echogenicity	43	36.40
Hyper echogenicity	17	14.40
No Neovascularization	38	32.20
Neovascularization I°	33	28.00
Neovascularization II°	22	18.60
Neovascularization III°	16	13.60
Neovascularization IV°	4	3.40
Tendon thickness origin (mm)	5.43 ± 0.92	
Tendon thickness thickest part (mm)	2.65 ± 0.65	

**Fig. 3:**
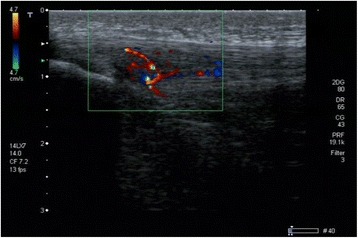
Sonographical findings in a player of the U-17 team with acute PTS in the right knee. Structural changes like hypo-echogenic areas; neovascularization and tendon thickening were all detectable

In addition, 63.6% of all players showed signs of neovascularization Fig. 3 of grade I – IV while only 32.2% did not show any signs of neovascularization Table 4. In contrast, neovascularizations of grade III and IV were found in only 20 players who all sustained a symptomatic PT.

**Table 4:** Comparison of VISA-*P* values in total and specific subscales divided by acute PTS syndrome, demonstrating significant lower scores for players sustainig acute PTS

	No- PTS	PTS	*P*-value
Visa P1	9.92 ± 0.37	7.73 ± 2.63	0.00
VISA P2	9.92 ± 0.29	9.13 ± 1.13	0.00
VISA P3	9.88 ± 0.73	8.86 ± 2.01	0.00
VISA P4	9.77 ± 0.85	6.87 ± 3.11	0.00
VISA P5	9.50 ± 1.38	6.06 ± 2.42	0.00
VISA P6	9.76 ± 0.75	7.8 ± 2.43	0.00
VISA P7	9.99 ± 0.10	8.8 ± 2.21	0.00
VISA P8	30.00 ± 0.00	21.53 ± 9.61	0.00
VISA total	98.77 ± 2.88	76.80 ± 18.65	0.00

## Discussion

To sum up the baseline data of this case-control study, 13.4% of all players of a German elite youth soccer academy sustained an acute PT.

Thus, the prevalence of PT was relatively high in youth elite players compared to the available data of adult players. Players at the age of 15 to 17 endure a risk almost twice as high as the other age groups of the youth academy players examined. The onset of the symptoms took place during training in 85.7% of all the cases and the players affected displayed local pain, pain at stretching, and significantly larger tendon diameters, hypo-echogenic areas, and neovascularization in the clinical and US examination. Taking into consideration a history of PT in the past, 27% of all players had sustained PT during their career with a medium stay off training and competition of 4.83 ± 6.78 months. Data of PT in the past is in accordance with data published by Hägglund et al. for adult champions and elite league players while the prevalence of acute PT was almost six times higher for youth soccer players examined in the present study [6].

The prevalence of 13.4% observed is in accordance with 11% in Swedish elite youth volleyball players, but remarkably higher than 7% reported for male and female basketball players at the age of 14–16 years and lower than 36% observed for adult professional volleyball players [2, 18, 19]. These differences most likely represent the wide variance of tendon load in different sport disciplines with a higher prevalence’s in explosive jumping sports. Therefore, volleyball and soccer can be considered high risk disciplines for tendon pathologies [20].

Regarding risk factors, position, cleats, pitch surface etc. were examined. Several authors identified a high amount of training hours as a significant risk factor in professional adult players [8, 21]. In the present study training amount did not correlate with the onset of PT, which is in accordance with the findings of Lian et al. [22]. Furthermore, BMI, height, leg dominance, shoes, and pitch surface did not correlate either. In contrast, age was a significant risk factor with the U-17 players having the highest risk for developing PT (Odds ratio 1.89). The fact that a well examined risk factor like training hours could not be confirmed in the present cohort might be due to the fact that the cohort examined presented a wide range of training hours with 3.5 h in the U-13 and up to 20 h in the U-23 (mean 10.35 ± 3.86 h weekly).. Nonetheless, age seems to be a remarkably important risk factor. Mersman et al. observed that elite youth volleyball players at their mid-adolescence showed adult-like characteristics of the quadriceps muscle. At the same time those players displayed a relative deficit of the patellar tendon, resulting in high level of stress during maximum contraction [23]. In accordance with the present data, Couppe et al. assumed that these high levels of tendon stress found in mid-age athletes might have an impact on tendon injury. As described by several authors, higher tendon stiffness is induced by hypertrophy as a result of the tendon’s process of adaption to higher stress [24]. This slow adaptive process due to low tendon metabolism is supposed to compensate the unfavorable relation of muscular strength and tendon loading capacity. This finally leads to different temporal dynamics of muscle and tendon adaption [25].

US is a valid method to verify these structural changes [11]. In the present study, all players with clinical symptoms of PT also had structural changes such as hypo-echogenic areas and neovascularization of grade III-IV. Additionally, another 30 players showed hypo-echogenic areas and another seven players were diagnosed with neovascularization of grade III-IV resulting in 43/232 tendons with structural changes (18.53%). These findings are in accordance with Gisslen et al. [18], Cook et al. who observed similar changes in 71/268 patellar tendons (26%) in junior elite basketball players [2], and 18% (18/98 patellar tendons) by Fredberg et al. [3]. In accordance with the present study, Cook et al. also found a higher prevalence of PT in the oldest third of the basketball players examined. They hypothesized that the combination of a slower metabolism in older players and the delayed onset of the synthesis of collagen type I more than 48 h after exercise might result in a higher risk of damage to the tendon [2]. Longitudinal studies are required to examine the importance of these structural changes in the mid- to long term follow-up period on the one hand and the potential of specific preventive programs on the other.

The importance of tendon adaption to higher loads is underlined by the significant increase of tendon diameter at the thickest point in players with PTS (4.58 ± 0.98 mm vs. 4.0 ± 0.54 mm, *p* = 0.001). Tendon thickening has been proven to be an indicator of tendinopathy [15]. In healthy adults, tendon thickness of the patellar tendon varies between 3 and 5 mm [26]. Comparable data of adolescents is rare. Recently, Cassel et al. examined patellar and Achilles sport-specific tendon thickness in 500 adolescent competitive athletes. In this study a general tendon adaption of different age groups to sport-specific landing could not be proven. Nonetheless, their values of 3.9 ± 0.5 mm for players older than 13 years of age in the high-risk sport soccer were comparable to the present cohort. Thus, patellar tendons of athletic children already show thickness values similar to those of adult athletes.

### Limitations

It has to be taken into consideration that youth elite soccer players all playing at the highest level of their age groups are rarely examined in current literature. Nonetheless, the study lacks a control group of non-youth elite soccer players. Additionally, age groups are not heterogeneous as there are always two teams in the U-13, U15, and U-17 in contrast to only one U-19 and U-23 team in the academy. Furthermore, only male athletes were examined. As mentioned before, players were not controlled for sexual maturity (i.e. “Tanner stages” or hormone blood tests) and a one-stage examination was performed evaluating baseline data only. Finally operator dependence of US examination has not to be considered as potential limitation as all examinations were performed by one experienced and licensed investigator (AH). Longitudinal investigations will be required to verify the importance of the structural changes detected.

## Conclusion

Prevalence of patellar tendinopathy in youth elite soccer is relatively high when compared to available date from adult players. Especially players at the age of 15 to 17 are at risk. Tendon thickening, hypo-echogenic areas and neovascularization are more common in symptomatic PTS.

## Abbreviations

BMI: Body-mass-index
DEGUM: Deutsche Gesellschaft für Ultraschall in der Medizin
PTS: Patella **t**endon **s**yndrom
ROI: Region of interest
U-13/23: Under 13 years of age
US: Ultrasound
Visa P: Victorian Institute of Sports Assessment Patellar
